# Cost Utility of cognition-enhancing interventions for individuals with first-episode psychosis: a naturalistic evaluation

**DOI:** 10.1186/s12962-021-00292-6

**Published:** 2021-07-01

**Authors:** Nicholas J. K. Breitborde, Emily K. Bell, Cindy Woolverton, Jacob G. Pine, Heather Waslter, Aubrey M. Moe

**Affiliations:** 1 Department of Psychiatry and Behavioral Health, The Ohio State University, Columbus, USA; 2 Department of Psychology, The Ohio State University, Columbus, USA; 3 56th Medical Group, Luke Air Force Base, Glendale, USA; 4 Department of Psychology, University of Arizona, Tucson, USA

**Keywords:** Cognition, Coordinated specialty care, Cost utility, First-episode psychosis, Cognitive remediation

## Abstract

**Background:**

Although effective treatments are available to address the cognitive deficits experienced by individuals with first-episode psychosis, provision of such treatments within Coordinated Specialty Care (CSC) programs is rare. One factor that may contribute to this is uncertainty about the cost implications of providing cognitive-enhancing treatments within the American mental healthcare system. The aim of this study is to complete a naturalistic evaluation of the cost utility of incorporating two different cognitive-enhancing interventions within an American CSC program.

**Methods:**

Participants included 66, predominately white (75.38%), individuals with first-episode psychosis (19 women and 47 men) with a mean age of 22.71 years. Quality adjusted life years (QALYs) and cost of care were tracked among these individuals during their participation in a CSC program. These data were compared among three groups of participants during their first six months of care: (i) individuals who participated in metacognitive remediation therapy (MCR), (ii) individuals who participated in computerized cognitive remediation (CCR), and (iii) individuals who participated in no cognitive-enhancing intervention.

**Results:**

Participation in MCR, but not CCR, was associated with larger gains in QALYs than participation in no cognitive-enhancing intervention within a CSC program. Moreover, data support the cost utility of MCR as compared to CCR or no-cognitive enhancing intervention within a CSC program. Conversely, CCR did not appear to be a cost-effective addition to CSC services.

**Conclusions:**

Our results highlight the potential cost utility of incorporating MCR within CSC programs for individuals with first-episode psychosis. However, given study limitations, these results should be interpreted cautiously until replicated by large, randomized controlled trials.

*Trial Registration* ClinicalTrials.gov Identifier NCT01570972, registered April 4, 2012, Retrospectively registered, https://clinicaltrials.gov/ct2/show/NCT01570972?term=breitborde&draw=2&rank=6.

## Background

Efforts to identify effective strategies to mitigate the morbidity and mortality experienced by individuals with first-episode psychosis (FEP) have highlighted the benefits of multicomponent, team-based intervention provided early in the course of these illnesses. Within the United States, this intervention strategy has been named “Coordinated Specialty Care” (CSC)—a multicomponent treatment package comprised of individual psychotherapy, family psychoeducation, medication management, and supported employment and education [1]. Recent trials have demonstrated the clinical benefits (e.g., reduced symptomatology and improved functional outcomes) and feasibility of delivering CSC within the American mental health system [2–4]. These data, in combination with recent federal legislation providing dedicated funding via increased Substance Abuse and Mental Health Services Administration Mental Health Block Grants, have sparked an unprecedented dissemination of CSC throughout the United States [5].

Yet, despite the benefits of CSC, there is still room for improvement with regard to treatment outcomes for individuals with first-episode psychosis [FEP: 6, 7]. In response to these findings, Kline and Keshavan [8] have suggested that the current iteration of CSC may represent a foundational set of interventions on which other interventions may be added to further improve outcomes among individuals with FEP that are not currently address by CSC. One such outcome currently not directly addressed within this foundational set of CSC interventions is cognitive functioning. Declines in cognitive functioning are nearly ubiquitous among individuals with psychotic disorders [9] and are recognized as a rate-limiting factor in their recovery efforts [10]. Data from the RAISE Early Treatment Program trial indicate that receipt of CSC is not associated with gains in cognitive functioning that exceed those associated with receipt of usual care among individuals with FEP [11].

Although efficacy of cognition-enhancing treatments in promoting improvements in cognitive functioning among individuals with psychosis is well-documented [12], including for individuals with first-episode psychosis specifically [13–15], provision of such interventions within American CSC programs is rare [5]. One factor that may contribute to this scarcity is uncertainty with regard to the cost implications of providing cognition-enhancing treatments [16]. Although available data suggest that cognition-enhancing treatments may be cost-effective [17–19], these data come solely from studies completed outside of the United States and may not be representative of the economic performance of these interventions within the American healthcare system. Moreover, no study to date has examined the economic performance of such interventions among individuals with FEP, specifically.

Thus, the goal of this study is to examine the cost utility of incorporating two cognition-enhancing interventions (i.e., drill-and-practice computerized cognitive remediation and metacognitive remediation therapy) within the existing clinical services in an American CSC program.

## Methods

Analyses reported as part of this manuscript were part of a larger project approved by the University of Arizona Institutional Review Board (IRB Project Number: 09-1113-02), and all study procedures conform with the Declaration of Helsinki. Written informed consent was obtained from all participants 18 years or older. For individuals younger than 18 years, written informed consent was obtained from the participant’s legal guardian and written assent was obtained from the participant.

### Participants

Sixty-six individuals with FEP were recruited from the Early Psychosis Intervention Center [EPICENTER: 20]. Eligibility criteria for EPICENTER include: (i) diagnosis of a schizophrenia-spectrum disorder or affective disorder with psychotic features as per the Structured Clinical Interview for the DSM-IV-TR [21]; (ii) < 5 years since the initial onset of psychotic symptoms [22] as determined using the Symptom Onset in Schizophrenia Inventory [23]; (iii) being between the ages of 15–35; and (iv) premorbid IQ > 70 as estimated using the Reading Subtest of the Wide Range Achievement Test [24]. Of note, as highlighted elsewhere, the term “first-episode psychosis” is typically operationally defined to capture individuals early in the course of a psychotic disorder as opposed to individuals who are experiencing psychotic symptoms for the first time [22]. Demographic data for study participants are reported in Table 1.

**Table 1:** Baseline demographic data

	Participants (*N* = 66)
Age (years; M ± SD)	22.71 ± 4.13
Gender	19 women; 47 men
Race	
White	75.38% (50/66)
Multiracial	16.92% (11/66)
African American	3.07% (2/66)
Asian	1.53% (1/66)
Native American	1.53% (1/66)
Native Hawaiian/Pacific Islander	1.53% (1/66)
Ethnicity	
Hispanic/latinx	29.23% (20/66)
Not hispanic/latinx	70.77% (46/66)
Median duration of psychotic illness	13.18 months
Diagnosis	
Schizophrenia-spectrum disorder	70.77% (46/66)
Affective disorder with psychotic features	29.23% (20/66)

### Procedures

#### Coordinated specialty care

Available interventions for EPICENTER participants include individual and group cognitive behavioral therapy for psychosis [25] and a modified version of multifamily group psychoeducation [26] tailored specifically for FEP [27]. Medication management was delivered by providers located in the larger outpatient clinic in which EPICENTER was situated or by community providers. Treatment planning at EPICENTER follows a shared decision-making model that is guided, in part, by data from a baseline assessment of symptomatology, real-world functioning, and cognition. Consequently, this study is best conceptualized as a naturalistic evaluation as we measured costs and service utilization as they unfolded as part of usual clinical care as opposed to experimentally manipulating what treatments (including cognition-enhancing treatments) that participants received.

#### Cognition-enhancing interventions

Between the opening of EPICENTER in early 2010 to mid-April 2014, 20 individuals with FEP selected to participate in a cognition-enhancing intervention during their first-six months of care at EPICENTER. As MCR had yet to be developed at the time EPICENTER opened, the first 10 individuals who opted to participate in a cognitive enhancing intervention received computerized cognitive remediation. Following the development of MCR, the second 10 individuals who selected to participate in a cognitive enhancing treatment received MCR. Both MCR and CCR were provided by masters and doctoral level clinicians with no between-intervention difference in level of provider. The remaining 46 individuals enrolled during this period did not participate in a cognition-enhancing intervention during their first six months of care at EPICENTER.

*Computerized cognitive remediation (CCR)* Individuals participating in drill-and-practice CCR completed computerized activities included in the PSSCogRehab program [28]. Exercises in this program target four domains of cognitive functioning: foundational skills (e.g., attention and processing speed), visual-spatial abilities, memory, and problem-solving. Participants begin with easier exercises and, once mastered, progress onto more difficult activities. A priori guidelines were utilized to ensure that the order of exercises and passing criteria for exercises were the same across participants.

During the first six-months of care, individuals participating in CCR were scheduled to complete two, 45–60 min sessions per week for a total of 52 sessions. A masters or doctoral-level clinician was present at all sessions to assist with the use of the computer software and address any concerns raised by the participant.

*Metacognitive remediation therapy (MCR) *MCR is among the growing number of individual psychotherapies whose primary therapeutic aim is improving metacognitive abilities [29] and builds upon existing strategies for improving metacognition included in psychotherapeutic and educational interventions (e.g., 30, 31]. Individuals participating in MCR completed two, 45–60 min psychotherapy sessions per week for a total of 52 sessions over the six-month follow-up Metacognitive skill development exercises included in MCR address four key therapeutic targets: (i) knowledge of cognition (i.e., knowledge of how, when, and why to use specific problem-solving strategies); (ii) regulation of cognition (i.e., the ability to select appropriate problem-solving strategies and monitor their effectiveness during and after implementation); (iii) intervening variables hypothesized to moderate the effective use of metacognitive skills in real world situations (i.e., arousal awareness and regulation; emotional awareness and regulation; and self-efficacy/motivation); and (iv) transfer of skills mastered in MCR sessions to real-world situations. Instruction in these skills is complemented by in-session practice of the skills using computerized activities to facilitate mastery before implementation in real-world settings. Exercises are personalized to address the specific intervening variables that may interfere with an individual’s ability to apply the skills learned in session to real-world situations. Results from previous research demonstrate that MCR produces improvements in metacognitive functioning among individuals with FEP [32] with additional downstream benefits on cognitive, social, and educational/occupational functioning that exceeded those produced by CCR [15].

### Measures

#### Cost assessments

Costs associated with the experience of FEP stem from a variety of sources including direct mental health services, contact with the legal system, utilization of societal benefits due to lack of employment or participation in school, and financial support from family members. Consequently, we opted to utilize a societal perspective for our analyses in which we assessed costs across all of these different domains during the six-month period in which participants completed cognitive remediation. These included intervention and treatment costs (i.e., cognitive-enhancing intervention costs, medication costs, and costs of other outpatient and inpatient mental health services), direct non-medical costs (i.e., costs incurred by family members of individuals with first-episode psychosis), and indirect costs (i.e., costs associated with non-participation in work/school and contact with the legal system). To remain consistent with our previous cost-analysis of this cohort [20], all costs were adjusted to 2015US$ values using the Consumer Price Index Inflation Calculator [33].

Utilization data for psychiatric care were quantified using the Service Utilization and Resources Form for Schizophrenia (SURF: 34]. Costs for use of inpatient psychiatric hospitalization were calculated using local cost data from the State of Arizona [35]. Costs for participation in MCR or computerized cognitive remediation included the cost of the PSSCogRehab software [28] as well as the cost of the therapist’s time. With regard to computer hardware, all therapists used their office computers to deliver these treatments. As these computers are provided to each therapist in the clinic regardless of whether they deliver MCR or computerized cognitive remediation, we did not include these costs in the analyses. The cost of therapist time associated with the delivery of MCR and computerized cognitive remediation was calculated by multiplying the number of sessions for each respective intervention (i.e., 52) by the sum of (i) the average hourly salary of a substance abuse and behavioral disorder counselor ($22.69) and (ii) average cost of benefits per hour worked for individuals working in health care and social assistance ($9.32) as reported in the 2015 National Compensation Survey [36]. The same formula was used to quantify costs of any non-EPICENTER outpatient mental healthcare. Antipsychotic medication use was assessed using the Current Medication Form [37]. Costs of antipsychotic medications were quantified using 2015 prices for non-generic medication. Costs associated with legal system contact were calculated by multiplying the number of legal system contacts captured by the SURF with the average cost of contact with the legal system for individuals with serious mental illness [38]. Participation in work or school was assessed using the Social Functioning Scale [39]. Costs stemming from unemployment and not participating in competitive educational activities were calculated using data from Corporation for National and Community Service and the White House Council for Community Solutions [40] and included costs associated with lost wages and poor health as well as savings due to lower utilization of government funds to facilitate participate in higher education. Financial support provided by family members was obtained from the SURF.

#### Quality adjusted life years (QALYs)

Health-related quality of life was assessed using the RAND 36-Item Health Survey [41] at enrollment in EPICENTER and after six-months of participation in EPICENTER services. Scores on the RAND 36-Item Health Survey were mapped onto the SF-6D [42] and then converted to QALYs using the nonparametric Bayesian method developed by Kharroubi and colleagues [43]. QALY values range from 1 (i.e., a year with perfect health) to 0 (i.e., a health state equivalent to death).

### Analysis

Results from Little’s missing completely at random test were consistent with the conclusion that the missing data were missing at random. Consequently, missing data were addressed using multiple imputation as per existing statistical recommendations [44].

Investigation of possible between-group differences in baseline characteristics among participants in the three intervention groups were completed using ANOVAs for continuous variables and Fisher’s Exact test for categorical variables. Longitudinal changes in QALYs were evaluated using linear regression with post-intervention QALYs included as the dependent variable, intervention group membership as the independent variable, and pre-intervention QALYs included as a covariate.

Cost data in our sample were positively skewed. As such, accelerated bias-corrected 95% confidence intervals for between-group mean differences (*M*_*diff*_) calculated via non-parametric bootstrapping were utilized to determine statistical significance of cost differences between the MCR, CCR, and no cognition-enhancing treatment groups using the protocol outlined by Burton et al. [45]. More specifically, between-group differences in mean costs for which the 95% confidence interval did not contain zero were considered to be statistically significant between-group differences.

**Fig. 1:**
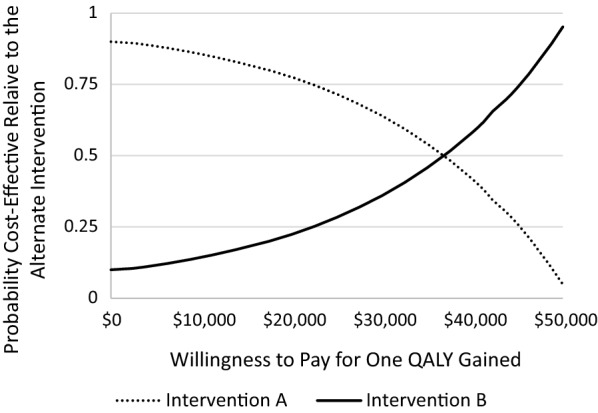
Example cost-effetiveness acceptabilty curve for two hypothetical interventions

Cost utility was evaluated via the construction of cost-effectiveness acceptability curves (CEAC). CEACs offer a graphical depiction the relationship between the probability that one intervention is cost-effective relative to another intervention (y-axis) across a range of values that a decision-maker would be willing to pay for a one-unit changed in a desired outcome variable [x-axis; 46]. Unlike cost-effectiveness planes which highlight the cost-effectiveness of an intervention at a single cost-effectiveness threshold (i.e., a single value that a decision-maker would be willing to pay to produce a one-unit change in a desired outcome), CEACs provide greater information with regard to the uncertainty associated with cost-effectiveness analyses by depicting the cost-effectiveness of an intervention across a range of cost-effectiveness thresholds [47]. An example CEAC is depicted in Fig. 1. In this example, if a stakeholder was willing to pay $0 to gain one QALY, the probability that Intervention A is cost-effective (relative to Intervention B) is 0.90. Conversely, at this value per QALY gained, the probability that Intervention B is cost-effective (relative to Intervention A) is 0.10. The curves for Intervention A and B intersect at $37,000 per QALY, indicating that in situations in which an individual is willing to pay >$37,000 per QALY gained, the probability that Intervention B is cost-effective exceeds the probability that Intervention A is cost-effective.

## Results

### Between-group baseline comparisons

Analyses revealed no baseline between-group difference in QALYs, the MATRICS Consensus Cognitive Battery [48] cognitive composite score, duration of psychotic illness, gender, race, ethnicity, diagnosis (i.e., schizophrenia-spectrum versus mood disorder with psychotic features, or age (all *p-*values > 0.23) among individuals participating in CCR, MCR, or no cognition enhancing intervention. Likewise, there was no between-group difference in the total cost of care during the 6-months prior to EPICENTER enrollment.

### QALYs

Pre- and post-intervention QALYs per group are displayed in Table 2. Participants in each respective intervention group experienced an increase in QALYs over the course of the study (all *p*-values < 0.05). Converting these gains into within-subject effect sizes (*d*_*av*_) revealed that individuals in the MCR group experienced the greatest gain in QALYs (*d*_*av*_ = 1.23), followed by the CCR group (*d*_*av*_ = 0.76) and the no cognitive enhancing intervention group (*d*_*av*_ = 0.34). Individuals in the MCR group (*t* = 3.04; *p* < 0.01), but not the computerized cognitive remediation group (*t* = 1.63; *p* = 0.11), experienced a greater increase in QALYs than individuals in the no cognition-enhancing intervention group. There was no statistically significant difference in the increase in QALYs over the intervention period between participants in the MCR group and CCR group (*t* = 0.97; *p* = 0.35).

**Table 2:** Per person costs and outcome data for the MCR, CCR, and no cognition-enhancing intervention groups (2015US$)

	MCR *M (SD)*	CCR *M (SD)*	No Cognition-Enhancing Intervention *M (SD)*
Inpatient care	$4,775 ($12,252)	$13,199 ($23,072)	$11,644 ($22,676)
Contact with the legal system	$6,443 ($18,168)	$3,220 ($8,686)	$2,653 ($9,130)
Costs associated with unemployment/not in school	$2,190 ($2,624)	$1,095 ($2,186)	$3,138 ($2,185)
Family costs	$1,384 ($3,292)	$1,424 ($2,962)	$351 ($2,972)
Outpatient care	$2,503 ($4,893)	$1,805 ($2,070)	$848 ($1,425)
Medication costs	$3,213 ($3,611)	$4,216 ($2,571)	$2,439 ($2,975)
Cognition-enhancing intervention costs	$1,830	$1,830	–
Total costs^a^	$22,336 ($21,351)	$26,787 ($27,231)	$21,070 ($26,116)
QALYs (pre-intervention)	0.50 (0.12)	0.57 (0.12)	0.57 (0.12)
QALYs (post-intervention)	0.69 (0.19)	0.68 (0.17)	0.62 (0.17)

^a^Values for individual cost items do not sum exactly to total costs due to rounding

### Cost data

There was no difference in total cost of care between the three treatment groups during the 6-month intervention period. With regard to the specific components of total cost, individuals in the CCR group had higher medication costs (*M*_*diff*_ = $1003; *95% CI* = $144 to $3398) and lower costs associated with unemployment/not being in school (*M*_*diff*_ = − $1095; *95% CI* = − $3147 to − $147) than individuals in the no cognition-enhancing group. There were no other between-group differences in specific components of total cost of care.

### Cost-effectiveness analyses

**Fig. 2:**
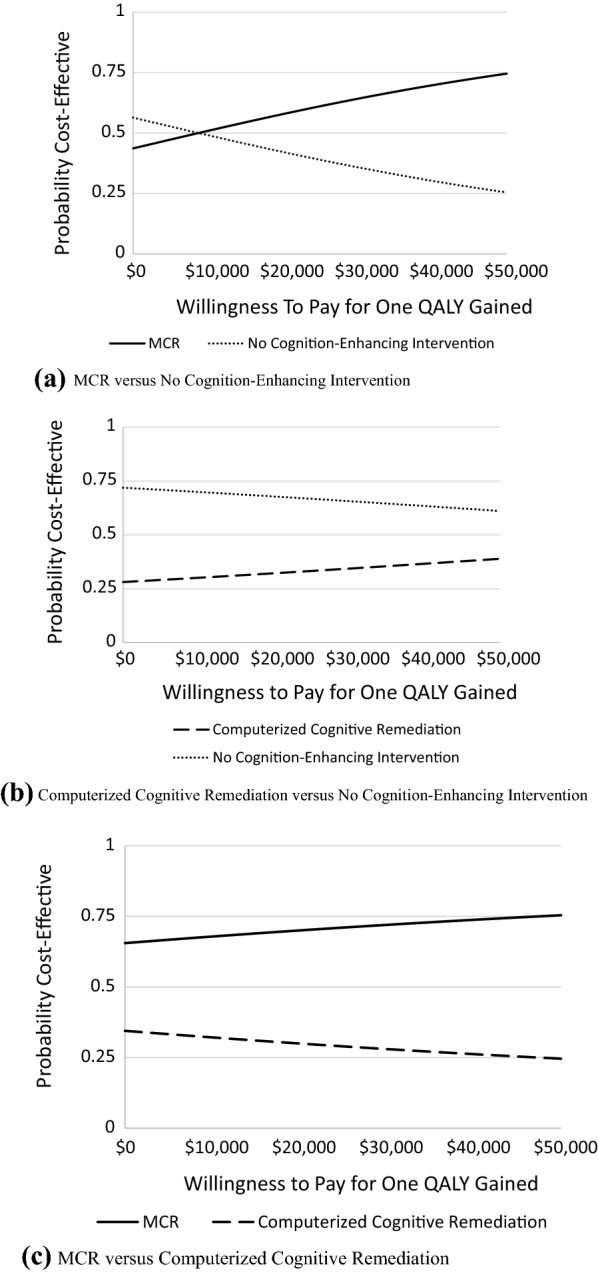
Cost-effetiveness acceptabilty curves for intervention group comparisons. **a** MCR versus no cognition-enhancing intervention. **b** Computerized cognitive remediation versus no cognition-enhancing intervention. **c** MCR versus computerized cognitive remediation

CEACs depicting the willingness to pay for the increase of one QALY are shown in Fig. 2. For all willingness-to-pay values ≥ $9,000 per one QALY increase, the probability that MCR was cost-effective exceeded the probability that participation in no cognitive-enhancing intervention was cost-effective (Fig. 2a). Conversely, the probability that CCR was cost-effective relative to no cognition-enhancing intervention never exceed 0.39 even for willingness-to-pay values of up to $50,000 (Fig. 2b). When compared to CCR, the probability that MCR was cost effective ranged from 0.65 at a willingness-to-pay value of $0 per QALY gained to 0.76 at a willingness-to-pay value of $50,000 per QALY gained (Fig. 2c).

## Discussion

The results of the current study highlight the potential cost benefits of incorporating MCR within multi-component treatment programs for individuals with FEP. Individuals with FEP who participated in MCR in addition to the other EPICENTER interventions and individuals who did not participate in a cognition-enhancing intervention both experienced increases in QALYs over the first six-months of EPICENTER services. However, individuals participating in MCR experienced larger gains in QALYs, and the probability that participating in MCR was cost-effective exceeded the probability that participation in no cognition-enhancing intervention was cost-effective in situations in which individuals were willing to pay ≥ $9,000 per QALY gained. This was well under the frequently used cost-effectiveness cut-off of US$50,000 per QALY gained [49] and compares favorably with available cost-effectiveness data on CSC. For example, in the seminal RAISE Early Treatment Program trial, Rosenheck and colleagues [50] found that at a willingness-to-pay value of $210,000 per QALY the probability that the NAVIGATE CSC intervention package was more cost-effective than usual care was 0.90–0.95. Comparatively, within the current study there was a 0.90–0.95 probability that MCR in combination with the other EPICENTER interventions was more cost-effective than participation in the other EPICENTER interventions alone at a value of $105,300 to $162,800 per QALY gained.

Conversely, data with regard to drill and practice CCR are less encouraging. Individuals in the CCR group experienced an increase in QALYs that did not differ in magnitude from the gain in QALYs experienced by individuals in the no cognition-enhancing intervention group. These data add to the growing evidence highlighting the limitations of drill and practice computerized cognitive remediation. For example, in their meta-analyses of cognitive remediation studies in schizophrenia, Wykes and colleagues [12] found that supplementing drill and practice cognitive remediation with training in strategies to improve performance produced greater gains in psychosocial functioning that drill and practice cognitive remediation alone.

Of note, this study did suffer from several limitations, including the lack of randomization to intervention condition and a small sample size—especially in the computerized cognitive remediation and MCR groups. With regard to the former, although there were no baseline group differences with regard to diagnosis, duration of illness, QALYs, baseline cognition, gender, race, ethnicity, or cost of care over the previous 6 months, it is possible that other, unmeasured variables may have differed between the intervention groups. Likewise, cost of computers was not included in the analyses as these are a standard resource provided to all therapist regardless of whether they provide cognition-enhancing treatments or not. Finally, data with regard to number of sessions of CCR and MCR completed were not tracked among participants. Thus, it is unclear whether treatment dose may have affected the results. Consequently, the results of the study should be interpreted cautiously until validated by data from larger, randomized controlled studies.

In total, these results highlight the possibility for continued enhancement of CSC programs for individuals with FEP. Metacognitive remediation therapy, but not drill-and-practice cognitive remediation, may be a cost-effective intervention that further enhances outcomes experienced by individuals participating in specialized care for FEP. As CSC programs continue to be rapidly established, continued efforts to develop new treatments may facilitate further improvements in the care provided to individuals with FEP in the United States.

## Conclusions

Declines in cognitive functioning are nearly ubiquitous among individuals with psychotic disorders [9] and are recognized as a rate-limiting factor in their recovery efforts [10]. Although effective cognition-enhancing treatments for individuals with psychosis have been identified [12], provision of such interventions within American CSC programs is rare [5]. One factor that may contribute to this scarcity is uncertainty with regard to the cost implications of providing cognition-enhancing treatments [16]. Our results highlight that some (i.e., MCR) but not all (i.e., CCR) cognition-enhancing interventions may be cost-effective additions within American CSC programs. However, given study limitations, these results should be interpreted cautiously until replicated by large, randomized controlled trials.

## Data Availability

The datasets generated and/or analyzed during the current study are not publicly available due to participants not providing consent to do so but are available from the corresponding author on reasonable request.

## Abbreviations

CCR: computerized cognitive remediation (drill-and-practice)
CEAC: Cost-effectiveness acceptability curve
CSC: Coordinated specialty care
FEP: First-episode psychosis
MCR: Metacognitive remediation therapy
QALY: Quality-adjusted life year
SURF: Service Utilization and Resources Form

## References

[1] Azrin ST, Goldstein AB, Heinssen RK (2016). Expansion of coordinated specialty care for first-episode psychosis in the US. Focal Point.

[2] Dixon LB, Goldman HH, Bennett ME, Wang Y, McNamara KA, Mendon SJ (2015). Implementing coordinated specialty care for early psychosis: the RAISE Connection Program. Psychiatric Services.

[3] Srihari VH, Tek C, Kucukgoncu S, Phutane VH, Breitborde NJK, Pollard J (2015). First-episode services for psychotic disorders in the US public sector: a pragmatic randomized controlled trial. Psychiatric Serv.

[4] Kane JM, Robinson DG, Schooler NR, Mueser KT, Penn DL, Rosenheck RA (2016). Comprehensive versus usual community care for first-episode psychosis: 2-year outcomes from the NIMH RAISE Early Treatment Program. Am J Psychiatry.

[5] Breitborde NJK, Moe AM (2017). Early intervention in psychosis in the United States: from science to policy reform. Policy Insights Brain Behav Sci.

[6] McGrath JJ (2012). The early intervention debate provides a distraction from another ‘unspeakable truth’. Aust N Z J Psychiatry.

[7] Breitborde NJK, Moe AM, Ered A, Ellman LM, Bell EK (2017). Optimizing psychosocial intervention in first-episode psychosis: current perspectives and future directions. Psychol Res Behav Manag.

[8] Kline E, Keshavan M (2017). Innovations in first episode psychosis interventions: The case for a “RAISE-Plus” approach. Schizophr Res.

[9] Keefe RS, Eesley CE, Poe MP (2005). Defining a cognitive function decrement in schizophrenia. Biol Psychiat.

[10] Green MF (1996). What are the functional consequences of neurocognitive deficits in schizophrenia?. Am J Psychiatry.

[11] Schooler N (2016). Cognitive functioning in first episode psychosis. Early Intervent Psychiatry.

[12] Wykes T, Huddy V, Cellard C, McGurk SR, Czobor P (2011). A meta-analysis of cognitive remediation for schizophrenia: methodology and effect sizes. Am J Psychiatry.

[13] Lee R, Redoblado-Hodge M, Naismith S, Hermens DF, Porter M, Hickie I (2013). Cognitive remediation improves memory and psychosocial functioning in first-episode psychiatric out-patients. Psychol Med.

[14] Saleem MM, Harte MK, Marshall KM, Scally A, Brewin A, Neill JC (2014). Positive effects of a novel cognitive remediation computer game (X-Cog) in first episode psychosis: a pilot study. Psychosis.

[15] Breitborde NJK, Woolverton C, Dawson SC, Bismark AW, Bell EK, Bathgate CJ (2017). Metacognitive skills training enhances computerized cognitive remediation outcomes among individuals with first-episode psychosis. Early Intervent Psychiatry.

[16] Breitborde NJK, Maple AM, Bell EK, Dawson SC, Woolverton C, Harrison-Monroe P, et al. Activity-regulated cytoskeleton-associated protein predicts response to cognitive remediation among individuals with first-episode psychosis. Schizophr Res. 2017;78:147–9. 10.1016/j.schres.2016.12.005 27989644

[17] Garrido G, Penadés R, Barrios M, Aragay N, Ramos I, Vallès V (2017). Computer-assisted cognitive remediation therapy in schizophrenia: Durability of the effects and cost-utility analysis. Psychiatry Res.

[18] Patel A, Knapp M, Romeo R, Reeder C, Matthiasson P, Everitt B (2010). Cognitive remediation therapy in schizophrenia: cost-effectiveness analysis. Schizophr Res.

[19] Wykes T, Reeder C, Williams C, Corner J, Rice C, Everitt B (2003). Are the effects of cognitive remediation therapy (CRT) durable? Results from an exploratory trial in schizophrenia. Schizophr Res.

[20] Breitborde NJK, Bell EK, Dawley D, Woolverton C, Ceaser A, Waters AC (2015). The Early Psychosis Intervention Center (EPICENTER): Development and six-month outcomes of an American first-episode psychosis clinical service. BMC Psychiatry.

[21] First MB, Spitzer RL, Gibbon M, William JBW (2002). Structured Clinical Interview for DSM-IV-TR Axis I Disorders, Research Version, Patient Edition (SCID-I/P).

[22] Breitborde NJK, Srihari VH, Woods SW (2009). Review of the operational definition for first-episode psychosis. Early Intervention in Psychiatry.

[23] Perkins DO, Leserman J, Jarskog LF, Graham K, Kazmer J, Lieberman JA (2000). Characterizing and dating the onset of symptoms in psychotic illness: The Symptom Onset in Schizophrenia (SOS) Inventory. Schizophr Res.

[24] Wilkinson GS, Robertson GJ (2006). Wide range achievement test (WRAT4).

[25] Breitborde NJK, Moe AM (2016). Cognitive behavioral therapy for people with first-episode psychosis: a service delivery protocol.

[26] McFarlane WR, Lukens E, Link B, Dushay R, Deakins SA, Newmark M (1995). Multiple-family groups and psychoeducation in the treatment of schizophrenia. Arch Gen Psychiatry.

[27] Breitborde NJK (2015). Family psychoeducation for first-episode psychosis: treatment protocol.

[28] Bracy O, PSSCogRebab (2012). Version 2012.

[29] Lysaker PH, Gagen E, Moritz S, Schweitzer RD (2018). Metacognitive approaches to the treatment of psychosis: a comparison of four approaches. Psychology Research Behavior Management.

[30] Schraw G (1998). Promoting general metacognitive awareness. Instr Sci.

[31] Nezu AM, Nezu CM, D’Zurilla T (2013). Problem-solving therapy: A treatment manual.

[32] Breitborde NJK, Moe AM (2016). Metacognitive remediation therapy: A service delivery protocol.

[33] United States Bureau of Labor Statistics. Consumer Price Index Inflation Calculator. United States Department of Labor; 2015. http://www.bls.gov/data/inflation_calculator.htm. Accessed 9 March 2015

[34] Rosenheck RA, Leslie DL, Sindelar J, Miller EA, Lin H, Stroup TS (2006). Cost-effectiveness of second-generation antipsychotics and perphenazine in a randomized trial of treatment for chronic schizophrenia. Am J Psychiatry.

[35] Arizona Department of Health Services. Emergency room visits and discharges of inpatients with mental disorders (ICD-9-CM codes 290–319) by category of first-listed and all-listed diagnoses and primary pays. 2012. http://www.azdhs.gov/plan/hip/for/mental/2012/mental912.xls. Accessed 20 March 2015.

[36] U. S. Bureau of Labor Statistics. National Compensation Survey: NCS Databases. 2017. https://www.bls.gov/ncs/data.htm. Accessed 6 April 2017

[37] Srihari VH, Phutane VH, Ozkan B, Chwastiak L, Ratliff JC, Woods SW (2013). Cardiovascular mortality in schizophrenia: Defining a critical period for prevention. Schizophr Res.

[38] Clark RE, Ricketts SK, McHugo GJ (1999). Legal system involvement and costs for persons in treatment for severe mental illness and substance use disorders. Psychiatric Serv.

[39] Birchwood M, Smith J, Cochrane R, Wetton S, Copestake S, The Social Functioning Scale (1990). The development and validation of a new scale of social adjustment for use in family intervention programmes with schizophrenic patients. Br J Psychiatry Suppl.

[40] Belfield CR, Levin HM, Rosen R (2012). The economic value of opportunity youth.

[41] Hays RD, Sherbourne CD, Mazel RM (1993). The RAND 36-Item Health Survey. Health Econ.

[42] Brazier J, Roberts J, Deverill M (2002). The estimation of a preference-based measure of health from the SF-36. J Health Econ.

[43] Kharroubi SA, Brazier JE, Roberts J, O’Hagan A, Modelling (2007). SF-6D health state preference data using a nonparametric Bayesian method. Journal of Health Economics.

[44] Graham JW (2009). Missing data analysis: Making it work in the real world. Annu Rev Psychol.

[45] Burton A, Billingham LJ, Bryan S (2007). Cost-effectiveness in clinical trials: using multiple imputation to deal with incomplete cost data. Clin Trails.

[46] Fenwick E, Byford S (2005). A guide to cost-effectiveness acceptability curves. Br J Psychiatry Suppl.

[47] Barton GR, Briggs AH, Fenwick EA (2008). Optimal cost-effectiveness decisions: the role of the cost-effectiveness acceptability curve (CEAC), the cost-effectiveness acceptability frontier (CEAF), and the expected value of perfection information (EVPI). Value Health.

[48] Nuechterlein KH, Green MF, Kern RS, Baade LE, Barch DM, Cohen JD (2008). The MATRICS Consensus Cognitive Battery, Part 1: Test selection, reliability, and validity. Am J Psychiatry.

[49] Grosse SD (2008). Assessing cost-effectiveness in healthcare: history of the $50,000 per QALY threshold. Expert Rev PharmacoEcon Outcomes Res.

[50] Rosenheck RA, Leslie D, Sint K, Lin H, Robinson DG, Schooler NR (2016). Cost-effectiveness of comprehensive, integrated care for first episode psychosis in the NIMH RAISE Early Treatment Program. Schizophr Bull.

